# MRI adipose tissue segmentation and quantification in R (RAdipoSeg)

**DOI:** 10.1186/s13098-022-00913-x

**Published:** 2022-10-08

**Authors:** Christine Haugen, Vegard Lysne, Ingfrid Haldorsen, Erling Tjora, Oddrun Anita Gudbrandsen, Jørn Vegard Sagen, Simon N. Dankel, Gunnar Mellgren

**Affiliations:** 1 Mohn Nutrition Research Laboratory, Department of Clinical Science, University of Bergen, Bergen, Norway; 2 Hormone Laboratory, Department of Medical Biochemistry and Pharmacology, Haukeland University Hospital, Bergen, Norway; 3 Centre for Nutrition, Department of Clinical Science, University of Bergen, Bergen, Norway; 4 Department of Heart Disease, Haukeland University Hospital, Bergen, Norway; 5 Mohn Medical Imaging and Visualization Centre (MMIV), Department of Radiology, Haukeland University Hospital, Bergen, Norway; 6 Section for Radiology, Departement of Clinical Medicine, University of Bergen, Bergen, Norway; 7 Department of Paediatrics, Haukeland University Hospital, Bergen, Norway; 8 Present Address: Faculty of Biosciences, University of Life Sciences, Ås, Norway

**Keywords:** Obesity, Subcutaneous adipose tissue, Visceral adipose tissue, MRI, Segmentation, Adipose tissue volume

## Abstract

**Background:**

Excess adipose tissue is associated with increased cardiovascular and metabolic risk, but the volume of visceral and subcutaneous adipose tissue poses different metabolic risks. MRI with fat suppression can be used to accurately quantify adipose depots. We have developed a new semi-automatic method, RAdipoSeg, for MRI adipose tissue segmentation and quantification in the free and open source statistical software R.

**Methods:**

MRI images were obtained from wild-type mice on high- or low-fat diet, and from 20 human subjects without clinical signs of metabolic dysfunction. For each mouse and human subject, respectively, 10 images were segmented with RAdipoSeg and with the commercially available software SliceOmatic. Jaccard difference, relative volume difference and Spearman’s rank correlation coefficients were calculated for each group. Agreement between the two methods were analysed with Bland–Altman plots.

**Results:**

RAdipoSeg performed similarly to the commercial software. The mean Jaccard differences were 10–29% and the relative volume differences were below ( ±) 20%. Spearman’s rank correlation coefficient gave p-values below 0.05 for both mouse and human images. The Bland–Altman plots indicated some systematic and proporitional bias, which can be countered by the flexible nature of the method.

**Conclusion:**

RAdipoSeg is a reliable and low cost method for fat segmentation in studies of mice and humans.

**Supplementary Information:**

The online version contains supplementary material available at 10.1186/s13098-022-00913-x.

## Background

The study of different adipose tissue depots has become highly important with the mounting evidence that visceral adipose tissue (VAT) contributes to metabolic syndrome and type 2 diabetes, while a high proportion of subcutaneous adipose tissue (SAT) is associated with a better metabolic profile [1–4]. Several methods exist for analysing the volumes of VAT, such as ultrasound [5], bioelectrical impedance analysis [6], and dual energy X-ray absorptiometry [7]. However these methods only give an indirect assessment of volume and often with poor accuracy. Computed tomography (CT) and magnetic resonance imaging (MRI) give a direct measure of VAT with high accuracy. Furthermore, these modalities enable volume analysis of other fat depots including SAT. Due to ionizing radiation exposure, CT may not always be an option in research studies. MRI does not involve ionizing radiation but has the drawback that, unlike CT, there are no fixed units. Instead, each image shows a relative difference in signal intensity between different tissues. In spite of this, MRI has shown to be as reliable as CT for fat segmentation [8].

Over the last decade, different manual, semiautomatic and automatic tools and software for fat segmentation of MRI images have been developed [9–13]. Concomitantly, the use and popularity of R [14] has increased greatly. R is a free and open-source software for statistical analysis and graphics. The ever-expanding library of R packages, as well as the high compatibility with other programming languages and software, makes R a highly flexible and practical tool for a wide range of tasks, including data management, statistical modelling, and visualisation. There are several packages for image manipulation and handling of medical images. However, no R protocols or packages for adipose tissue segmentation of MRI images exist.

We have integrated available packages to create a semi-automatic method for fat segmentation of MRI images in R. To test the new method, RAdipoSeg, we performed segmentation of SAT and VAT on images from lean and obese mice and humans, and compared with results from manual segmentation of the same images with the commercially available and frequently used SliceOmatic software (Tomovision, Montreal, Canada). Compared to SliceOmatic, RAdipoSeg allows for fat segmentation of MRI images with minimal cost and high flexibility regarding organisms, scanning techniques, and type of fat depots.

## Methods

### Tissue imaging and clinical data sampling

Images were taken from wild-type control mice in 2 different diet studies on genetically modified male mice [15, 16]. The mice were fed either a control (low-fat, LF, 3.8 kcal/g) or high-fat (HF, 4.7 kcal/g) diet for 10 weeks. Further details are presented in Additional file 3. MRI was performed in the kidney area with a 7 T small animal magnetic resonance tomograph with ParaVision 5.1 software (Bruker Biospin MRI Gmbh, Billerica, Massachusetts, USA). A series of axial base images (“base image”) were acquired with T1-weighted Rapid Acquisition with Relaxation Enhancement (RARE), and fat suppressed images (“water image”) were acquired in the same spatial domain with Chemical Shift-selective Fat Suppression (CHESS). Water suppressed images (“fat image”) were acquired by subtracting the water images from the base images. The image size was 256 × 256 voxels, with each voxel measuring 0.156 × 0.156 × 1 mm. Both MRI and glucose tolerance tests were performed in the last 3 weeks of the diet period. For the CD1 mice, glucose tolerance tests were performed by feeding the mice 2 mg/g glucose orally via tube. Blood glucose was measured at 0, 15, 30, 60, 120 and 240 min. The C57BL/6 J mice had glucose injected intraperitoneally, and blood glucose was measured at 0, 15, 30, 60, 120 and 180 min.

Human images were acquired from healthy subjects by a 2-point Dixon sequence performed on a 1.5 T Siemens Avanto running Syngo MR B17 (Syngo, Siemens, Erlangen, Germany), giving two groups of images in the same spatial domain; with the water and fat spins in-phase or out-of-phase, which is used to generate water and fat images [17]. The image size was 320 × 240 voxels, with each voxel measuring 1.234 × 1.234 × 2.5 mm.

For each mouse and each human subject, respectively, 10 sequential images in the kidney area were selected for segmentation. The kidney is located in approximately the same area in all subjects within each species, and is therefore well suited as an anchor point when comparing adipose tissue volumes. An approximate midpoint of the kidneys was found where there was an equal number of images on each side with at least 1 kidney visible. The 10 sequential images selected for volume quantification were the 5 closest images on each side of this midpoint.

### R packages

**Fig. 1:**
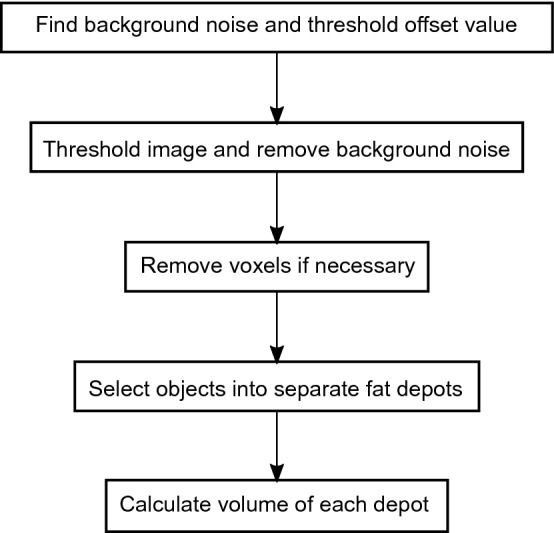
Overview of the workflow of fat segmentation of MRI images with RAdipoSeg. The procedure begin by finding and removing background noise, and thresholding the image. Removal of some voxels may be necessary to divide SAT from VAT, if the depots lie so close together that there is no line of black voxels between them in the image. Finally the objects are selected to the different depots and volume is calculated

This protocol was developed with R 3.6.3 in RStudio 1.2.5033 (RStudio, PBC, Boston, Massachusetts, USA). Functions are shown in cursive, and the package given in parentheses when not a basic R function. The package oro.dicom [18] was used for loading dicom files into R. Most of the functions used here are from EBImage [19] and imager [20]. The tidyverse [21] collection of packages were used for data handling and output formatting. A short description of fat segmentation is presented in Fig. 1. For a detailed protocol and further information about the packages, see Additional files 1, 2.

### Background noise and thresholding

The margins of each image were divided into 8 fields and the background noise calculated from randomly selected voxels from these fields. If some fields had high signal objects, e.g., arms, they were removed and the voxels were randomly selected from the remaining fields. The background noise (BG) was calculated as the sum of the mean and standard deviation (sd) of the voxel intensities. If objects with high signal intensity were not removed, the background noise was too high for use in further calculations.

Thresholding was used to separate objects representing adipose tissue from the rest of the image. Thresholding works by setting all voxels above or equal to a set signal intensity (the threshold) to 1 and all voxels below the threshold to 0. The threshold value was calculated from the voxel intensities along the contour of the body. The contour is the line of voxels which separates the body from the background in an MRI image. In an axial water suppressed image (fat image), a large portion of the voxels along the contour represent SAT. To find the contour, we used the base images for the mice and the in-phase images for the humans, which were thresholded using a modified version of Otsu’s method [22]. Then a contour finding algorithm was applied using *ocontour* (EBImage). The adipose tissue threshold for the fat images was found by subtracting sd from the mean of the intensities. When this value was below the calculated background noise, the threshold was recalculated as TC = BG + 0.1 × sd, where TC is the threshold, BG is the background noise and sd is the standard deviation of the voxel intensities, as described previously [10]. The fat images were then thresholded using *thresh* (EBImage). This function performs local thresholding using a moving rectangular window and TC as the offset value. The size of the rectangular window was optimized as 15 × 15 voxels for mouse images and 50 × 50 for human images.

The background noise was removed with a mask created using *fillHull* and *floodFill* (EBImage), which set all voxels outside of the mask to 0.

### Labelling image objects, manual editing, and volume estimation

Some images were edited manually for removal of areas (e.g., bone marrow) and then the images were labelled using *bwlabel* (EBImage). This function gives each piece of interconnected voxels a separate value, counting the objects. To distinguish SAT from VAT, it was necessary to divide some objects by manually setting voxels to 0, and then relabel the image. These voxels were automatically reinserted to the appropriate depot later to avoid loss of data. Voxels can also be manually added or deleted to correct for incomplete fat suppression caused by inhomogeneties in the magnetic field during scanning. However, this was not performed on these images. The selection of objects was achieved by using *grapPoint* (imager). New images were made with all SAT having voxel value 1 and VAT value 2, and the volumes were calculated by counting the voxels.

### Method evaluation

To evaluate the method, fat segmentation was performed on the same images with SliceOmatic’s region growing mode. Selections of the segmented images from both methods were validated by a radiologist. The volumetric overlap errors were calculated with the Jaccard distance [23], given as 100(1− (|A ∩ B|/|A ∪ B|), where 0% is a perfect overlap between segmentation by RAdipoSeg and SliceOmatic. The relative volume differences (RVD) between RAdipoSeg (A) and SliceOmatic reference (B) were calculated as 100((|A|− |B|)/|B|). Spearman’s rank correlation coefficients were calculated to test for linear correlation. Bland–Altman analyses were performed with differences between the methods on the y-axis and mean of the methods on the x-axis, and with 95% confidence intervals. Proportional bias was tested using Spearman’s rank correlation coefficients on the data from the Bland–Altman analyses. Normalcy was assessed by Shapiro–Wilk test. All statistical analyses and making of plots were conducted in R. The figures were created in Inkscape 1.0.0 (https://inkscape.org/), with text size being the only alteration made to the plots.

## Results

**Fig. 2:**
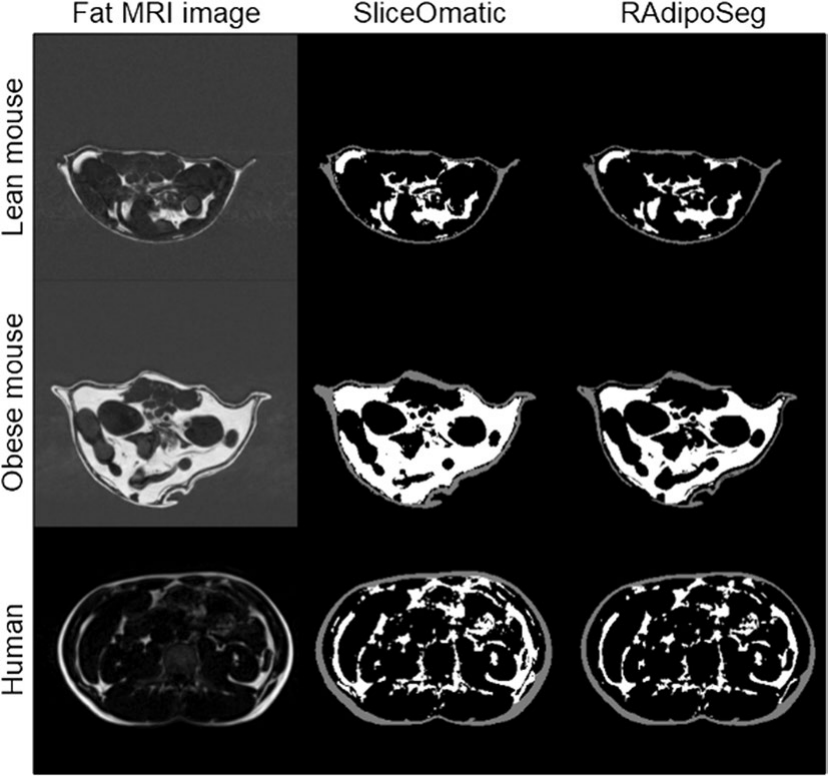
Comparison of fat segmentation by SliceOmatic and RAdipoSeg. One representative slice from each group of lean mice, obese mice and humans, shown from left to right as fat MRI image, SliceOmatic segmentation and RAdipoSeg segmentation. The grey background of the fat mouse MRI images show the high level of background noise

**Fig. 3:**
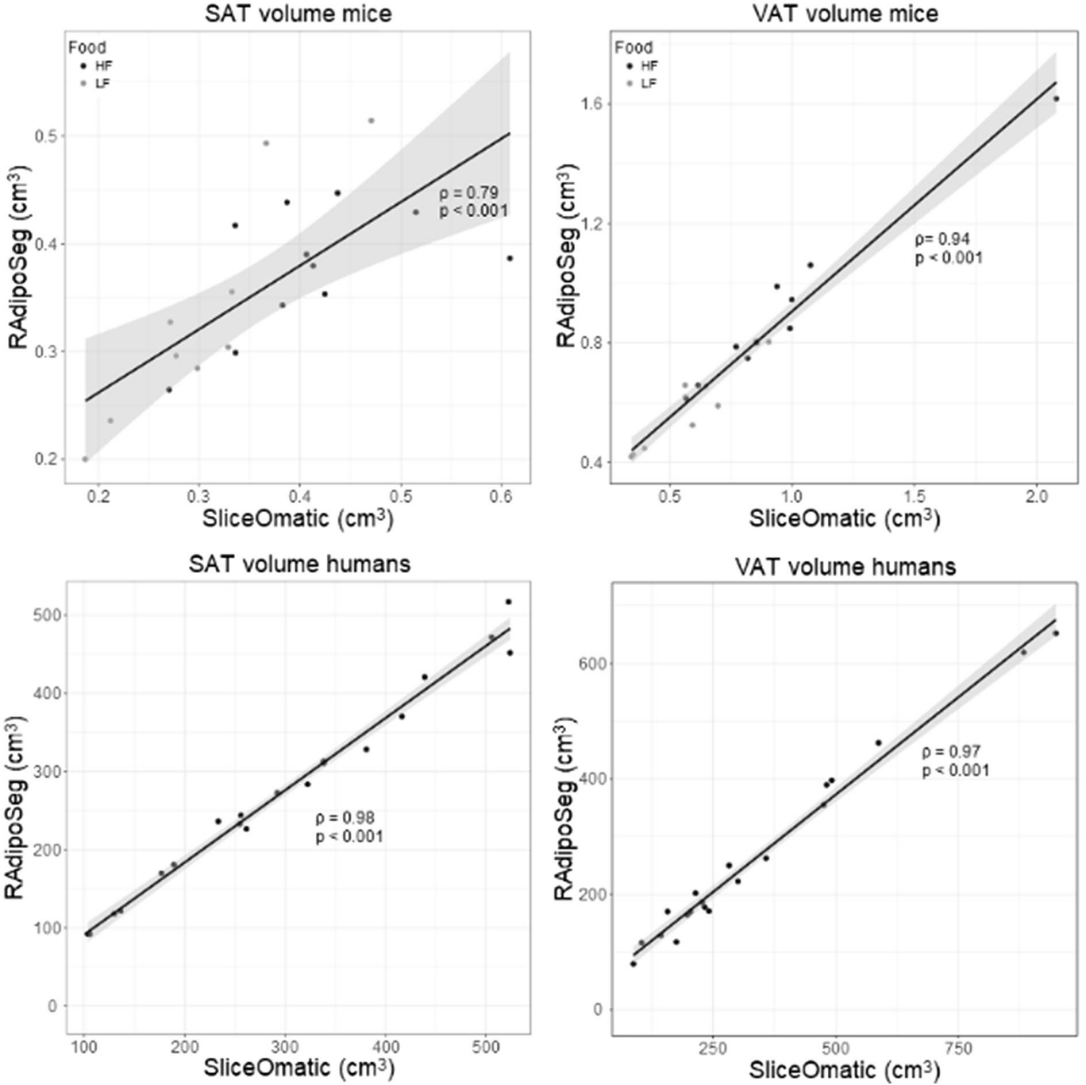
Test of linear correlation between the 2 methods for mice (n = 20) and humans (n = 20) using Spearman’s rank correlation coefficients. Volumes were calculated by summing the voxels from all images of each subject and multiplying with the voxel size. Data from the lean and obese mice were pooled together

**Fig. 4:**
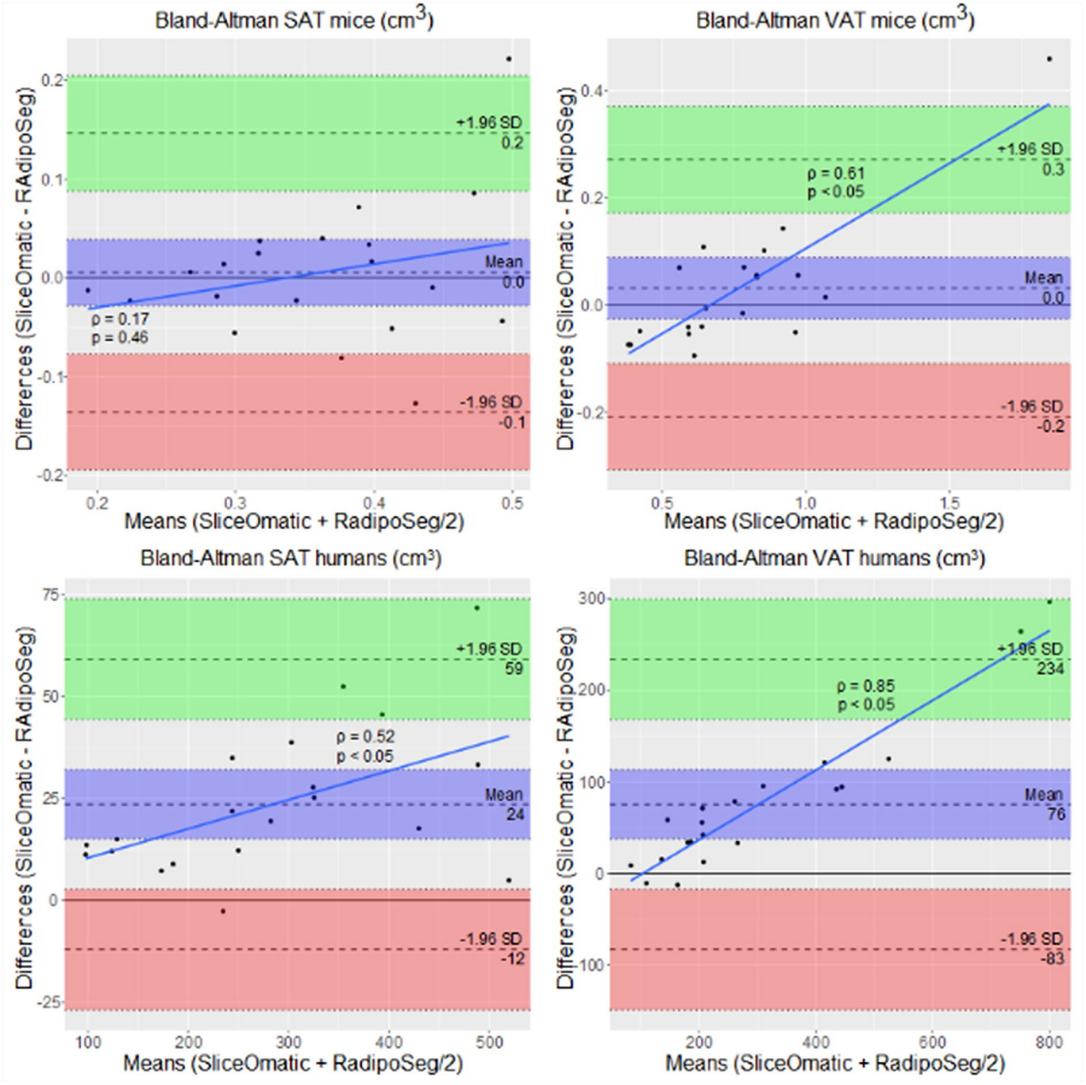
Bland–Altman plots of the volume in cm^3^ of VAT and SAT for mice (n = 20) and humans (n = 20), with 1.96 ×SD limits of agreement and 95% confidence interval. Volumes were calculated by adding the voxels from all images of each subject and multiplying with the voxel size. Data from the lean and obese mice were pooled together. Spearman’s rank correlation coefficients were calculated for estimation of proportional bias

Mouse images were pooled into two groups of lean and obese according to diet (an overview of the groups and clinical characteristics are shown in Additional file 3). The segmentation with RAdipoSeg took approximately the same time as with SliceOmatic, although images with medium high background noise took longer to segment with RAdipoSeg than images of good quality. Images with very high levels of background noise (9 mice) were difficult to segment by SliceOmatic and were excluded from the analysis. All the human images were of good quality and hence were included (clinical characteristics of the subjects are shown in Additional file 3). Representative images for each group are shown in Fig. 2, and a summary of results and statistical analyses are presented in Table 1. The Jaccard differences were below 26% in all cases except for SAT in lean mice, and RVDs were below ( ±) 20%. Both of these measures were higher for the lean mice compared to obese mice, which is expected since the differences in a lower fat volume will influence a proportional measure more strongly than the differences in a high fat volume. The RAdipoSeg tended to give a higher volume (positive RVD) for the lean mice and a lower volume (negative RVD) for humans compared with SliceOmatic. In general, Spearman statistics had a high linear correlation between the two methods for mice and humans (Table 1 and Fig. 3). The Bland–Altman analyses (Fig. 4) showed a systematic bias for the human images (p < 0.05), but not the mouse images. There was a proportional bias of higher differences with higher volume of VAT for both humans and mice (p < 0.05). This bias persisted in Bland–Altman plots performed with proportions (SliceOmatic—RAdipoSeg/means of both methods) on the y-axis (Additional file 4). Overall, RAdipoSeg gave a volume estimation similar to SliceOmatic, but with significant systematic and proportional bias.

**Table 1:** Jaccard differences, relative volume differences and Spearman’s rank correlation coefficients

	N	Jaccard difference (sd)			Relative volume difference (sd)			Spearman ρ (p-value)		
		SAT	VAT	TAT	SAT	VAT	TAT	SAT	VAT	TAT
Lean mice	9	28.9 (9.2)	21.2 (2.7)	23.0 (6.6)	12.5 (12.6)	7.4 (16.6)	8.6 (12.9)	0.88 (0.003)	0.75 (0.025)	0.97 (< 0.001)
Obese mice	11	23.6 (6.2)	16.8 (2.7)	18.2 (3.4)	− 3.9 (17.2)	− 2.9 (9.4)	− 3.1 (10.7)	0.48 (0.13)	0.95 (< 0.001)	0.87 (< 0.001)
All mice	20	26.0 (8.0)	18.8 (4.5)	20.3 (5.5)	3.5 (17.1)	1.7 (13.8)	2.2 (12.9)	0.79 (< 0.001)	0.94 (< 0.001)	0.94 (< 0.001)
Humans	20	10.4 (3.6)	22.2 (6.4)	15.4 (4.2)	− 8.2 (4.3)	− 17.7 (11.9)	− 12.9 (6.5)	0.98 (< 0.001)	0.97 (< 0.001)	0.99 (< 0.001)

Jaccard differences and Relative Volume Differences are expressed as mean (standard deviation). Spearman’s rank correlation coefficients were calculated on the volume in cm^3^ for each subject.

*SAT* Subcutaneus adipose tissue, *VAT* Visceral adipose tissue, *TAT* Total adipose tissue

When the glucose area under the curve (AUC, blood glucose mmol/L x min) was plotted against the number of pixels representing VAT (Additional file 5), there was a slightly increasing trend for the C57BL/6 J mice, showing a higher glucose AUC with higher number of VAT pixels. The CD1 mice showed no significant difference between the diet groups in body weight, fasting glucose and glucose AUC. However, it should be noted that the CD1 mouse strain is known to be more resistant to high-fat diets than several other mice strains [24].

## Discussion

In any study where adipose tissue volume is of interest, a reliable method for quantification is important. Equally important is the access to a flexible method that is available for researchers with limited funding studying any model organism. Here, we present a new method for adipose tissue segmentation and quantification, RAdipoSeg, and compare this with a frequently used, commercially available method. The 2 methods correlate well, even though the background noise in many of the mouse images caused a greater variance in this group compared to the human images.

RAdipoSeg has 2 advantages over SliceOmatic. First, SliceOmatic is an expensive software with a large annual fee in addition to a costly licence, while R is completely free. Second, RAdipoSeg sets the threshold by a standardised method, reducing subjectivity as a source of error, while the SliceOmatic threshold is set manually by the operator. This is especially important for the human images, which have a much lower range of signal intensities (below 500) compared with the mice images (around 30,000). With a lower range, a small change in the threshold will have a much larger effect. This is likely to have caused the systematic bias observed in the Bland–Altman plots of the human images; however, the bias may also have been caused by the automatic threshold set by RAdipoSeg being too high, rather than the manual threshold of SliceOmatic being set too low. A study with a larger sample size, which also looks into the inter- and intra-reproducibility of both methods, may answer this question.

For VAT we observed a proportionial bias for both human and mouse images. With a larger tissue volume there is also a larger region of interest, which increases the number of voxels that can be mislabelled. We would therefore expect more differences the larger the volume. However, when plotting proportional differences against the means the bias did not disappear. We also observed a higher Jaccard difference and RVD for VAT compared with SAT in humans. This and the proportional bias may be caused by the offset value used for thresholding with RAdipoSeg being based on the signal intensity of SAT. When there is a higher variation in the signal intensity of VAT compared with SAT, the adipose voxels with low signal in VAT could be excluded from segmentation. The selection of human subjects in this study was limited and consisted of metabolically healthy controls. In a study with more variation in visceral adipose tissue between the human subjects, the segmentation of individuals with a higher VAT volume may give an underestimated quantification of VAT. This could in turn underestimate the differences between the subjects in the study, and create a bias in the data towards a lower VAT volume. If the automatically generated threshold is seen as inadequate, only a minor alteration of RAdipoSeg is required for setting a manual threshold. This would prevent the underestimation of volume in subjects with a high level of adipose tissue, at the cost of an increase in subjectivity. In many studies, having a standardised method might be preferable to gaining a more exact adipose tissue volume estimation.

Image quality was a factor in comparing the two methods. The CHESS technique of fat suppression is known to be sensitive to B_0_ and B_1_ heterogeneity, which may be the reason for the incomplete fat suppression observed in many of the mouse images. It was difficult to segment images with high background noise using SliceOmatic. The threshold had to be set very low and the fat had to be subjectively separated from the background noise. RAdipoSeg gave good segmentation of these images, but lower image quality increased the duration of the procedure by requiring more manual interference. RAdipoSeg uses a local threshold, which allows for better separation between signal and background noise than a global threshold. Each voxel is analysed based on the neighbouring voxels, while global threshold is affected by artefacts in the entire image. Also, with RAdipoSeg, the noise outside of the region of interest is automatically deleted by the mask, which gives a clear boundary against the background. With SliceOmatic the boundary had to be decided manually during segmentation. In addition to the low sample size, this may be the reason for the poor Spearman’s correlation coefficient between the methods for SAT in obese mice. Image quality was not a factor for the human images, as they all had little background noise.

Only the region growing module of the SliceOmatic software could be used in this study, as the high level of background noise made the other modules impractical or impossible to use. For the human images, the other modules for fat segmentation offered by SliceOmatic were available and may have yielded a different result.

There are several automatic and deep learning software for fat segmentation of MRI images, such as FatSegNet [12] and an algorithm for MatLab (MathWorks, Natick, Massachusetts) [13]. However, most of these are specialized for human images and, in some cases, for specific human regions. Others require a high number of images for training. For smaller studies on images from rodents, an automatic method designed for human images may not be easily adapted. In comparison, RAdipoSeg is flexible and can be used on any MRI images from any organism. By implementing other programming languages, e.g., Python, RAdipoSeg can be further automatised, thereby improving speed and usability of the procedure (Additional files 6 and 7).

There was a slightly increasing trend for the correlation between VAT volume and blood glucose AUC for the C57BL/6 J mice. Since ectopic accumulation of lipids in the liver is an important predictor for glucose intolerance [25], it should be noted that RAdipoSeg may also be adapted to measure liver adiposity. Liver percentage fat fraction can be measured using the method Iterative decomposition of water and fat with echo asymmetry and least squares estimation (IDEAL) [26]. RAdipoSeg may be able to pick out the geographical position of the liver from MRI images where the liver, or the immediate surrounding tissues, have a high pixel intensity. The geographical location can then be used to measure percentage liver fat fraction from the corresponding IDEAL MRI images.

## Conclusions

We present and validate a novel method for fat segmentation of MRI images, RAdipoSeg, in the free and open-source software R. A comparison with the SliceOmatic software shows that RAdipoSeg can be used to give a reliable and consistent volume estimate of fat in studies of mice and humans. Though a proportional bias was detected for images with a high adipose tissue volume, this can be countered by setting the threshold manually. Furthermore, though the fat suppression technique will affect the results to some degree, this new method can be used on images acquired either by the CHESS or the 2-point Dixon technique. RAdipoSeg is therefore a suitable and reliable method for relative comparisons of fat depot images from studies with standardised imaging.

## Supplementary Information

**Additional file 1: **can be viewed in RStudio (https://www.rstudio.com/) Complete code for performing adipose tissue segmentation with RAdiposeg. This format allows for convenient and direct use of the code in RStudio.

**Additional file 2.** Complete code for performing adipose tissue segmentation with RAdiposeg, read format. This format allows for reading the entire code without RStudio.

**Additional file 3.** Characteristics of the mouse and human studies/groups used for the fat segmentation.

**Additional file 4.** Bland-Altman plots with proportions on the y-axes. Plots calculated with volume in cm^3^ of VAT and SAT for mice (n=20) and humans (n=20), with 1.96 ×SD limits of agreement and 95 % confidence interval. Volumes were calculated by adding the voxels from all images of each subject and multiplying with the voxel size. Data from the lean and obese mice were pooled together. Spearman’s rank correlation coefficients were calculated for estimation of proportional bias.

**Additional file 5.** Blood glucose AUC plotted against VAT volume. The plot was separated by methods, RAdipoSeg and Sliceomatic, and mouse strain, CD1 and C57BL/6J. Test of linear correlations were performed using Spearman’s rank correlation coefficients. P-values (rho) for the CD1 mice were 0.84 (− 0.10) for RAdipoSeg and 0.93 (0.05) for SliceOmatic. P-values (rho) for the C57BL/6J mice were 0.01 (0.79) for RAdipoSeg and 0.01 (0.78) for SliceOmatic.

**Additional file 6.** MRI image data. All data collected from each MRI image using the RAdipoSeg and SliceOmatic segmentation methods.

**Additional file 7.** Readme file with details about Additional file 6.

## Data Availability

All data generated and analysed during this study are included in this published article and its supplementary information files. The MRI images analysed during the current study are available from the corresponding author on reasonable request.

## Abbreviations

AUC: Area under the curve
BG: Background noise
BMI: Body mass index
BW: Body weight
CHESS: Chemical shift-selective fat suppression
HF: High-fat diet
IDEAL: Iterative decomposition of water and fat with echo asymmetry and least squares estimation
LF: Low-fat control diet
RARE: Rapid acquisition with relaxation enhancement
REK: Western norway regional committee for medical research ethics
RVD: Relative volume difference
SAT: Subcutaneous adipose tissue
TC: Threshold
VAT: Visceral adipose tissue
