# Supplementary material for: MRI adipose tissue segmentation and quantification in R (RAdipoSeg)
Source: Diabetol Metab Syndr. 2022 Oct 8;14:146. doi: 10.1186/s13098-022-00913-x (PMC9548171; doi:10.1186/s13098-022-00913-x)
Supplement: Supplementary file 2 — Additional file 2. Complete code for performing adipose tissue segmentation with RAdiposeg, read format. This format allows for reading the entire code without RStudio. [file 13098_2022_913_MOESM2_ESM.pdf]

MRI adipose tissue segmentation and quantification in R (RAdipoSeg)

Haugen C, Lysne V, Haldorsen I, Tjora E, Gudbrandsen OA, Sagen J, Dankel SN, Mellgren G

15 11 2021

Brief overview

This is a code for segmentation of adipose tissue from MRI images. The default is the segmentation of subcutaneous (SAT) and visceral (VAT) white adipose tissue, however the script can be used for the segmentation of any adipose tissue. The script was created to be used with images acquired with the CHESS method of fat suppression, and therefore includes code for creating water suppressed images from the base and fat suppressed images. The images are ordered into a tibble, and the majority of the functions are performed on all images at the same time. Only the manual steps must be performed on each image seperately.

The code was built in RStudio 1.2.5001 with R version 3.6.3. The packages used for image loading and processing were oro.dicom 0.5.3, imager 0.42.1 and EBImage 4.28.1. In addition, several packages from the tidyverse 1.3.0 collection were used for data handling and output formatting, including tibble 3.0.0, dplyr 0.8.5, tidyr 1.0.2, purrr 0.3.3 and rlang 0.4.5. All packages can be installed from CRAN, except for EBImage which is available via Bioconductor (https://bioconductor.org/packages/release/bioc/html/EBImage.html).

Load packages

```
library(oro.dicom)
library(imager)
library(EBImage)
library(tidyverse)
```

Functions

- `BGnoise`
  - Calculate background noise by randomly selecting voxels from 8 fields along the edge of the image
- `vis_bg_area`
  - Visualises the 8 fields used for calculating background noise. If any field contains high signal objects (such as artifacts or body parts), they may be excluded from the background noise calculation
- `contour_threshold`
  - Used for thresholding in the process of finding the contour of the Region of Interest (ROI) and making a mask
- `make_cdf`
  - A function extracting the first coordinates of the different objects in the image, used for making a helper image for selection of objects
- `selectobjects`
  - Selects objects for volume estimation

```r
#' Calculate background noise
#'
#' Randomly selects voxels from 8 fields along the edge of the image, and
#' returns the mean + sd of these voxels as a measure of background noise.
#'
#' @param image a matrix representing a MRI image
#' @param rows number of rows in image
#' @param cols number of columns in image
#' @param size size of the fields, in voxels from the edge. Defaults to 20
#' @param totvoxels total number of selected voxels, distributed between the
#' fields. Defaults to 500
#' @param ignore which fields to ignore
#'
#' @return
BGnoise <- function(image, rows, cols, size = 20, totvoxels = 500,
                    ignore = NULL){

  A1 <- image[1:(rows/2), 1:size]
  A2 <- image[(rows/2+1):rows, 1:size]
  A3 <- image[(rows-size+1):rows, size:(cols/2)]
  A4 <- image[(rows-size+1):rows, (cols/2):(cols-size)]
  A5 <- image[(rows/2+1):rows, (cols-size+1):cols]
  A6 <- image[1:(rows/2), (cols-size+1):cols]
  A7 <- image[1:size, (cols/2):(cols-size)]
  A8 <- image[1:size, size:(cols/2)]


  s <- tibble(data = list(A1, A2, A3, A4, A5, A6, A7, A8)) %>%
    rowid_to_column("id") %>%
    filter(!(id %in% ignore)) %>%
    mutate(voxels = as.integer(totvoxels/nrow(.)),
           randomvoxels = map2(data, voxels,
                               ~sample(.x, .y, replace = F))) %>%
    pull(randomvoxels) %>%
    unlist()

  mean(s) + sd(s)

}

#' Visualises the fields for calculation of background noise
#'
#' Draws a grid representing the 8 fields from which to randomly pick voxels
#' for the calculation of background noise. The fields are numbered from 1-8,
#' and fields containing artefacts may subsequently be ignored when calculating
#' the background noise using the BGnoise function
#'
#' @param image a matrix representing a MRI image
#' @param rows number of rows in image
#' @param cols number of columns in image
#' @param size size of the fields, in voxels from the edge. Defaults to 20
#'
#' @return
```

```r
vis_bg_area <- function(image, rows, cols, size = 20){

  mat <- matrix(0, rows, cols)
  mat[, c(size, cols-size)] <- 1
  mat[c(1:size, (rows-size + 1):rows),cols/2] <- 1
  mat[c(size, rows-size), size:(cols-size+1)] <- 1
  mat[rows/2, c(1:size, (cols-size):cols)] <- 1

  image(paintObjects(mat, normalize(toRGB(image))))
  text(c(20, (rows/2+20), (rows-size/2), (rows-size/2), (rows/2+20), 20,
         (size/2), (size/2)),
     c((size/2), (size/2), (size+20), (cols/2+20), (cols-size/2), (cols-size/2),
       (cols/2+20), (size+20)),
     c("1", "2", "3", "4", "5", "6", "7", "8"), col = "white")

}

#' Thresholding of image to find the contour of the ROI
#'
#' @param image a matrix representing a MRI image
#' @param yval the cutoff on the y-axis of the density function for locating
#' the maximum x-value (value for thresholding)
#'
#' @return
#' @export
#'
#' @examples
contour_threshold <- function(image, cutoff = 0.0001){
  max(density(image)$x[density(image)$y > cutoff])
}


#' Make CDF column
#'
#' @param image a labelled image (labelled with `EBImage::bwlabel()`)
#'
#' @return
#' @export
#'
#' @examples

make_cdf <- function(image){
  x <- image

  as.data.frame(table(image)) %>%
             slice(-1) %>%
             mutate(MRows = map_dbl(image,
                                  ~which(x == .x, arr.ind = T)[1,1]),
             MCols = map_dbl(image,
                             ~which(x == .x, arr.ind = T)[1,2]),
             image = as.character(image))
}
```

```r
#' Manually select objects for fat volume calculation
#' @param imagenumber which image on the list to select objects from
#' @param numberofobjects the maximum number of objects to select
#' numberofobjects must be increased if more than 30 is to be selected, if less,
#' keep default. When running function, exit the window when no more objects are
#' wanted.

selectobjects <- function(imagenumber, numberofobjects = 30){
  select <- vector(mode = "list", numberofobjects)
  for (i in 1:numberofobjects) {
  grabp <- grabPoint(as.cimg(rotate(result$bw[[imagenumber]],
                                    angle = 270)),
                     output = "value")
      if(attr(grabp, "coords")[[1]] == 0){
        select[i:numberofobjects] <- NaN
        break
  }
    select[[i]] <- grabp[[1]]
 }
  select
}
```

Work flow

Preliminary step

- Generate lists of images, one for finding contour and making mask, one for analysis or for creating images for analysis
- `subjectname` is the name of the subject that is being analysed

```r
Imagelist1 <- readDICOM("") # Stores the mask images in object "Imagelist1"
Imagelist2 <- readDICOM("") # Stores fat suppressed or water suppressed images
# in object "Imagelist2"

subjectname <- "example" # To be used when exporting results
```

Step 1

- Organises the images with some metadata in a tibble
- Select which images to use for creating contour and mask, and which images to analyse, by passing the corresponding column name to `Formask` and `Foranalyse`.

```r
result <- tibble::tibble(id = str_extract(names(Imagelist1$img), "[^/]*$"),
                 Rows = extractHeader(Imagelist1$hdr, "Rows"),
                 Cols = extractHeader(Imagelist1$hdr, "Columns"),
                 Base = Imagelist1$img, # Column containing base images
                 Fatsuppressed = Imagelist2$img, # Column containing fat
# suppressed images
                 Watersuppressed = map2(Base, Fatsuppressed, ~.x - .y), # Water
# suppressed images, made by subtracting fat suppressed images from base
# images, or
                 #Watersuppressed = Imagelist2$img # If loaded in directly
                 )
```

```
Formask <- rlang::quo(Base) # Select imagelist to be used for finding contour
# and creating mask
Foranalyse <- rlang::quo(Watersuppressed) # Select imagelist for analysis
```

Step 1a

- Explore one of the images to see whether some fields must be ignored when calculating background noise, using the `vis_bg_area()` function
- Plot density curves for all `Formask` images, to find appropriate cutoff for preliminary thresholding. Make sure the hline is above the second curve

```
vis_bg_area(result$Base[[1]], result$Rows[[1]], result$Cols[[1]], 20)

result %>%
  select(id, {{Formask}}) %>%
  mutate({{Formask}} := map({{Formask}}, ~(as.vector(.x)))) %>%
  unnest({{Formask}}) %>%
  ggplot(aes({{Formask}}, color = id)) +
  geom_density() +
  geom_hline(yintercept = 0.005) + # Adjust cutoff
  coord_cartesian(xlim = c(0, 1000)) # Zoom axis for readability
```

Step 1b

- Calculate background noise, ommit areas if necessary
- Calculate threshold based on the cutoff found in step 1a. The cutoff does not need to be accurate, only good enough to give ROI as one object without holes in the contour.

```
result <- result %>%
  mutate(BGnoise = pmap_dbl(list({{Foranalyse}}, Rows, Cols),
                            ~BGnoise(..1, ..2, ..3, size = 20, totvoxels = 500,
                                     ignore = NULL)), # Select fields to ignore
         Threshold = map_dbl({{Formask}},
                             ~contour_threshold(..1, cutoff = 0.0001)))
# Select appropriate cutoff here

result
```

Step 2

- `Formask` is thresholded with the threshold value found in step 1b, creating the binary image `t`.
- To find the contour of the ROI, artefacts and objects early in the image `t` must be removed by filtering out the objects with number of voxels < 1000. This can be altered if necessary.
- The position of the voxels around the edge of the image is calculated with the `EBImage::ocontour()` function, applied to `t`, and stored in a column called `con`.
- Check whether all the contours are captioned by plotting the contours

```
result <- result %>%
  mutate(t = pmap(list({{Formask}}, Rows, Cols, Threshold),
                  ~thresh(..1, ..2/2 - 10, ..3/2 - 10, offset = ..4)),
         t_table = map(t, ~as.data.frame(table(bwlabel(..1))) %>%
                         filter(Freq < 1000)),
         t_clean = map2(t, t_table,
                        ~rmObjects(bwlabel(..1),
```

```
                                    as.numeric(as.character(..2$Var1))) > 0.5),
         con = map(t_clean, ~ocontour(..1)))

map(result$con, ~plot(..1[[1]]))
```

Step 3

- The positions around the edge (`con`) is used to find the voxel intensities of SAT in the `Foranalyse` image (`int`).
- Offset value (`TC`) is calculated as mean - sd
- If TC is lower than `BGnoise`, a new TC is calculated as background noise + 0.1*sd
- TC can also be set manually
- Threshold the `Foranalyse` image (`tu`). Be aware that the width (w) and height (h) of `EBImage::thresh()` may need optimisation
- All the binary images are displayed for evaluation

```
result <- result %>%
  mutate(int = map2({{Foranalyse}}, con, ~..1[..2[[1]]]),
         TC = map_dbl(int, ~mean(..1) - sd(..1)),
         TC = case_when(TC < BGnoise ~ BGnoise + 0.1*sd(int[[1]]), TRUE ~ TC),
         #TC = 30, # Uncomment for manual TC
         tu = map2({{Foranalyse}}, TC, ~thresh(..1, w = 15, h = 15,
                                               offset = ..2)))

map(result$tu, ~EBImage::display(rotate(.x, angle = 270), method = "raster"))
```

Step 4

- A mask is generated from the contour calculated in step 2
- The mask is used to remove everything outside of the ROI (`ffm`)
- `ffm` is displayed for evaluation

```
result <- result %>%
  mutate(tu = map(tu, ~replace(..1, c(1,1), 0)),
         mask = pmap(list(con, Rows, Cols),
                     ~fillHull(replace(matrix(0,..2,..3), ..1[[1]], 1))*2),
         #ffm = tu # Uncomment for skipping the mask. Comment out the next line.
         ffm = map2(tu, mask, ~floodFill((..1 + ..2), c(1,1), col = 0,
                                        tolerance = 1) - ..2)
         )

map(result$ffm, ~EBImage::display(rotate(.x, angle = 270), method = "raster"))
```

Manual removal of unwanted voxels

- Use this if there are areas that should be removed in all images (e.g. bone marrow)
- May also be used to remove noise outside of ROI, instead of the mask

1. Each image is displayed in turn
2. Make a square covering the voxels to be removed
3. The voxels are automatically set to 0
4. Display for evaluation

```
for(i in seq_len(nrow(result))){
  grabl <- grabLine(as.cimg(result$ffm[[i]]), output = "coord")
```

```
  result$ffm[[i]][c(grabl[[1]]:grabl[[3]]), c(grabl[[2]]:grabl[[4]])] <- 0
  }

map(result$ffm, ~EBImage::display(rotate(.x, angle = 270), method = "raster"))
```

Step 5a

- Labelling the objects in `ffm` with `EBImage::bwlabel`
- Small objects can be filtered out. Set to 0 if all objects are to be kept at this stage
- Extract first coordinates of each object into `cdf` column with the `make_cdf` function

```
result <- result %>%
  mutate(bw = map(ffm, ~bwlabel(..1)),
         t = map(ffm, ~as.data.frame(table(bwlabel(..1))) %>%
                   filter(Freq <= 0)), # For removal of small objects of less
# than `Freq` voxels.
         bw = map2(ffm, t,
                   ~rmObjects(bwlabel(..1),
                              as.numeric(as.character(..2$Var1)))),
         cdf = map(bw, ~make_cdf(.x)))
```

Step 5b

- Create folder `Helper images`
- Save images makes it easier to find voxels that need editing and object selection

```
dir.create("Helper images")
im <- select(result, !!Formask)
for(i in 1:nrow(result)){

  img <- toRGB(Image(im[[1]][[i]]))
  pm1 <-  paintObjects(result$bw[[i]], normalize(img), opac=c(1,0), col="red")

  png(paste0("./Helper images/", subjectname, result$id[i], "helper.png"),
      height = 10, width = 20, res = 500, units = "cm")

  par(mfcol = c(1,2))
  EBImage::display(rotate(colorLabels(result$bw[[i]]), angle = 270),
                   method = "raster")
  text(c(result$cdf[[i]]$MCols), result$Rows[1]-c(result$cdf[[i]]$MRows),
       as.character(c(result$cdf[[i]]$image)), col="white")

  EBImage::display(rotate(pm1, angle = 270), method = "raster")

dev.off()
}
```

Manual alteration of voxels in individual images

- Optional step for:
  - Deleting voxels that have been mislabelled during scanning due to incomplete fat suppression

  - Separation of objects into SAT and VAT
    - Collect the coordinates in `coord_change` for adding the voxels to the approptiate fat depot in the final result

    - Try to keep all the deleted voxels from all images in one depot for ease of use
  - The `imager::grabLine()` function allows for selection of coordinates using mouse click on the appearing image
  - When this step has been completed for all images that need alteration, redo step 5

```
# Creation of a data frame for collection of coordinates
coord_change <- tibble(Imageno = numeric(),
                       x_coord1 = numeric(),
                       x_coord2 = numeric(),
                       y_coord1 = numeric(),
                       y_coord2 = numeric())


imagenumber <- 1 # Select image number to be edited

# Select coordinates and set voxels to 0
grabl <- grabLine(as.cimg(result$ffm[[imagenumber]]), output = "coord")
result$ffm[[imagenumber]][c(grabl[[1]]:grabl[[3]]),
                          c(grabl[[2]]:grabl[[4]])] <- 0

# Collect the coordinates in data frame. Must be done between each use of
# grabLine as grabl will change
coord_change <- bind_rows(coord_change, tibble(Imageno = imagenumber,
                                               x_coord1 = grabl[[1]],
                                               x_coord2 = grabl[[3]],
                                               y_coord1 = grabl[[2]],
                                               y_coord2 = grabl[[4]]))
```

Step 6 Manual object selection

- Use helper images to identify objects representing SAT or VAT
- Select SAT objects with the function `selectobjects`
- VAT objects can be assigned as all the remaining objects after SAT has been selected, or use `selectobjects` again
- Make list of lists with all the correctly assigned objects in all the images

```
image1_sat <- selectobjects(1)
image1_vat <- as.data.frame(table(result$bw[[1]])) %>%
  slice(-1) %>%
  select(Var1) %>%
  filter(!Var1 %in% image1_sat[!is.na(image1_sat)])
#image1_vat <- selectobjects(1)
image2_sat <- selectobjects(2)
image2_vat <- as.data.frame(table(result$bw[[2]])) %>%
  slice(-1) %>%
  select(Var1) %>%
  filter(!Var1 %in% image2_sat[!is.na(image2_sat)])
#image2_vat <- selectobjects(2)
image3_sat <- selectobjects(3)
image3_vat <- as.data.frame(table(result$bw[[3]])) %>%
  slice(-1) %>%
  select(Var1) %>%
  filter(!Var1 %in% image3_sat[!is.na(image3_sat)])
```

```r
#image3_vat <- selectobjects(3)
image4_sat <- selectobjects(4)
image4_vat <- as.data.frame(table(result$bw[[4]])) %>%
  slice(-1) %>%
  select(Var1) %>%
  filter(!Var1 %in% image4_sat[!is.na(image4_sat)])
#image4_vat <- selectobjects(4)
image5_sat <- selectobjects(5)
image5_vat <- as.data.frame(table(result$bw[[5]])) %>%
  slice(-1) %>%
  select(Var1) %>%
  filter(!Var1 %in% image5_sat[!is.na(image5_sat)])
#image5_vat <- selectobjects(5)
image6_sat <- selectobjects(6)
image6_vat <- as.data.frame(table(result$bw[[6]])) %>%
  slice(-1) %>%
  select(Var1) %>%
  filter(!Var1 %in% image6_sat[!is.na(image6_sat)])
#image6_vat <- selectobjects(6)
image7_sat <- selectobjects(7)
image7_vat <- as.data.frame(table(result$bw[[7]])) %>%
  slice(-1) %>%
  select(Var1) %>%
  filter(!Var1 %in% image7_sat[!is.na(image7_sat)])
#image7_vat <- selectobjects(7)
image8_sat <- selectobjects(8)
image8_vat <- as.data.frame(table(result$bw[[8]])) %>%
  slice(-1) %>%
  select(Var1) %>%
  filter(!Var1 %in% image8_sat[!is.na(image8_sat)])
#image8_vat <- selectobjects(8)
image9_sat <- selectobjects(9)
image9_vat <- as.data.frame(table(result$bw[[9]])) %>%
  slice(-1) %>%
  select(Var1) %>%
  filter(!Var1 %in% image9_sat[!is.na(image9_sat)])
#image9_vat <- selectobjects(9)
image10_sat <- selectobjects(10)
image10_vat <- as.data.frame(table(result$bw[[10]])) %>%
  slice(-1) %>%
  select(Var1) %>%
  filter(!Var1 %in% image10_sat[!is.na(image10_sat)])
#image10_vat <- selectobjects(10)

VAT_SAT <- list(list(VAT = image1_vat[!is.na(image1_vat)],
                     SAT = image1_sat[!is.na(image1_sat)]),
          list(VAT = image2_vat[!is.na(image2_vat)],
               SAT = image2_sat[!is.na(image2_sat)]),
          list(VAT = image3_vat[!is.na(image3_vat)],
               SAT = image3_sat[!is.na(image3_sat)]),
          list(VAT = image4_vat[!is.na(image4_vat)],
               SAT = image4_sat[!is.na(image4_sat)]),
          list(VAT = image5_vat[!is.na(image5_vat)],
```

```
             SAT = image5_sat[!is.na(image5_sat)]),
        list(VAT = image6_vat[!is.na(image6_vat)],
             SAT = image6_sat[!is.na(image6_sat)]),
        list(VAT = image7_vat[!is.na(image7_vat)],
             SAT = image7_sat[!is.na(image7_sat)]),
        list(VAT = image8_vat[!is.na(image8_vat)],
             SAT = image8_sat[!is.na(image8_sat)]),
        list(VAT = image9_vat[!is.na(image9_vat)],
             SAT = image9_sat[!is.na(image9_sat)]),
        list(VAT = image10_vat[!is.na(image10_vat)],
             SAT = image10_sat[!is.na(image10_sat)])
        )


# Check if the length of `VAT_SAT` corresponds to length of result table.
length(VAT_SAT) == nrow(result) # Should return TRUE
```

Step 6b

- Add `VAT_SAT` to `result`
- Make images containing SAT (voxel value 1) and VAT (voxel value 2) (`vox`)
- Add the voxels deleted during object division (stored in `coord_change`)

```
result <- result %>%
  mutate(VAT_objects = map(VAT_SAT, ~.x$VAT), SAT_objects = map(VAT_SAT,
                                                                ~.x$SAT))

result <- result %>%
  mutate(vox = map2(Rows, Cols, function(x,y) matrix(0, x, y)),
         vox = pmap(list(vox, bw, VAT_objects, SAT_objects),
                    function(vox, bw, VAT, SAT){
                      for(i in SAT){
                        vox[bw==i] <- 1
                        }
                      for(i in VAT){
                        vox[bw==i] <- 2
                        }
                      vox
                      }))

# Add in deleted voxels. All should be either SAT or VAT or more editing will
# be needed
for(i in seq_len(nrow(coord_change))){
  imagenumber <- coord_change[[i,1]]
  result$vox[[imagenumber]][c(coord_change[[i,2]]:coord_change[[i,3]]),
                           c(coord_change[[i,4]]:coord_change[[i,5]])] <- 2
# 1 for SAT, 2 for VAT
  }

map(result$vox, ~EBImage::display(rotate(normalize(.x), angle = 270),
                                  method = "raster"))
```

Useful codes for further manual editing

- Correction of mistakes made in earlier steps
- Adding voxels that are missing due to incomplete fat suppression

```
imagenumber <- 1

# Giving a view of where the objects in `vox` are on the water suppressed image
img <- toRGB(Image(result$Watersuppressed[[imagenumber]]))
pm1 <-  paintObjects(result$vox[[imagenumber]], normalize(img), opac=c(1,0),col="red")
# Edit by clicking on image
grabl <- grabLine(as.cimg(pm1), output = "coord")
result$vox[[imagenumber]][c(grabl[[1]]:grabl[[3]]),
                          c(grabl[[2]]:grabl[[4]])] <- 0

# For relabelling of a wrongly assigned object
bwspes <- bwlabel(result$vox[[imagenumber]])
grabl <- grabPoint(as.cimg(bwspes), output = "value")
result$vox[[imagenumber]][bwspes==grabl] <- 1 # 1 for SAT, 2 for VAT
```

Step 7

- Quantify VAT, SAT and TAT
- Make table
- Export results and images

```
result <- result %>%
  mutate(Count_SAT = map_dbl(vox,  ~sum(.x == 1)),
         Count_VAT = map_dbl(vox,  ~sum(.x == 2)),
         Count_TAT = Count_SAT + Count_VAT)

result <- result %>%
  unnest(c(id, BGnoise, TC, Count_SAT, Count_VAT, Count_TAT))

save_table <- result %>%
  mutate(Name = subjectname) %>%
  select(Name, `Imagenumber`= id,
         `Background noise` = BGnoise, `Threshold value` = TC,
         `Subcutaneous AT` = Count_SAT,
         `Visceral AT` = Count_VAT, `Total AT` = Count_TAT)

# Uncomment these lines to save results as csv
#write_csv2(save_table,
#           paste0(subjectname,
#                  "_segmentation_results.csv")) %>%
#  knitr::kable()

# Save the images
dir.create("Result images")
for(i in 1:nrow(result)){
  filename <- paste0("./Result images/", subjectname, "_", result$id[[i]],
                     "_SAT_VAT", ".png")
  writeImage(normalize(rotate(result$vox[[i]], angle = 270)), filename)
}
```
