# Supplementary material for: MRI adipose tissue segmentation and quantification in R (RAdipoSeg)
Source: Diabetol Metab Syndr. 2022 Oct 8;14:146. doi: 10.1186/s13098-022-00913-x (PMC9548171; doi:10.1186/s13098-022-00913-x)
Supplement: Supplementary file 3 — Additional file 3. Characteristics of the mouse and human studies/groups used for the fat segmentation. [file 13098_2022_913_MOESM3_ESM.pdf]

Additional file 3. Characteristics of the mouse and human studies/groups used for the fat segmentation.

					Average (SD)					
**Study**	**Organism**	**Diet**	**N (males)**	**Included**	**Age**	**Weight**	**BMI**	**Fasting gl**	**AUC**	**Liver w/bw**
Bjune and Haugen et al., 2019	CD1 mice	Control	8 (8)	6	15.7w (1.21)	35.0 (4.42)	NA	6.0 (0.62)*	1880 (444)*	0.043 (0.007)*
		HF	5 (5)	4	15.5w (0.58)	38.0 (5.61)	NA	4.6 (1.20)*	1638 (542)*	0.038 (0.004)*
Svärd et al., 2019	C57BL/6J mice	Control	8 (8)	3	21.0w (0.00)	27.5 (0.42)	NA	6.8 (1.10)	2700 (939)	NA
		HF	8 (8)	7	21.7w (1.60)	33.7 (1.95)	NA	8.4 (1.35)	3681 (567)	NA
Tjora et al., 2013	Human	NA	20 (10)	20	36.1y (16.6)	NA	24.6 (3.4)	NA	NA	NA

Diets are control diet with 3.8 kcal/g or high-fat (HF) diet with 4.7 kcal/g. Included is N after the exclusion of subjects with MRI images of poor quality. Mean and standard deviations (SD) are calculated from the included selection. Age is in weeks (w) for the mice and years (y) for the humans. Weight is in grams and body-mass index (BMI) in kg/cm^2. Fasting glucose (gl) is measured in mmol/L and area under the curve (AUC) in mmol/L x min. The AUC for the CD1 mice was measured with time points 0, 15, 30, 60, 120 and 240 min, and for the C57BL/6J mice with time points 0, 15, 30, 60, 120 and 180 min. Liver w/bw is liver weight in grams / body weight in grams.

* Data not included in the original publication
