# Supplementary figures and images for: MRI adipose tissue segmentation and quantification in R (RAdipoSeg)
Source: Diabetol Metab Syndr. 2022 Oct 8;14:146. doi: 10.1186/s13098-022-00913-x (PMC9548171; doi:10.1186/s13098-022-00913-x)

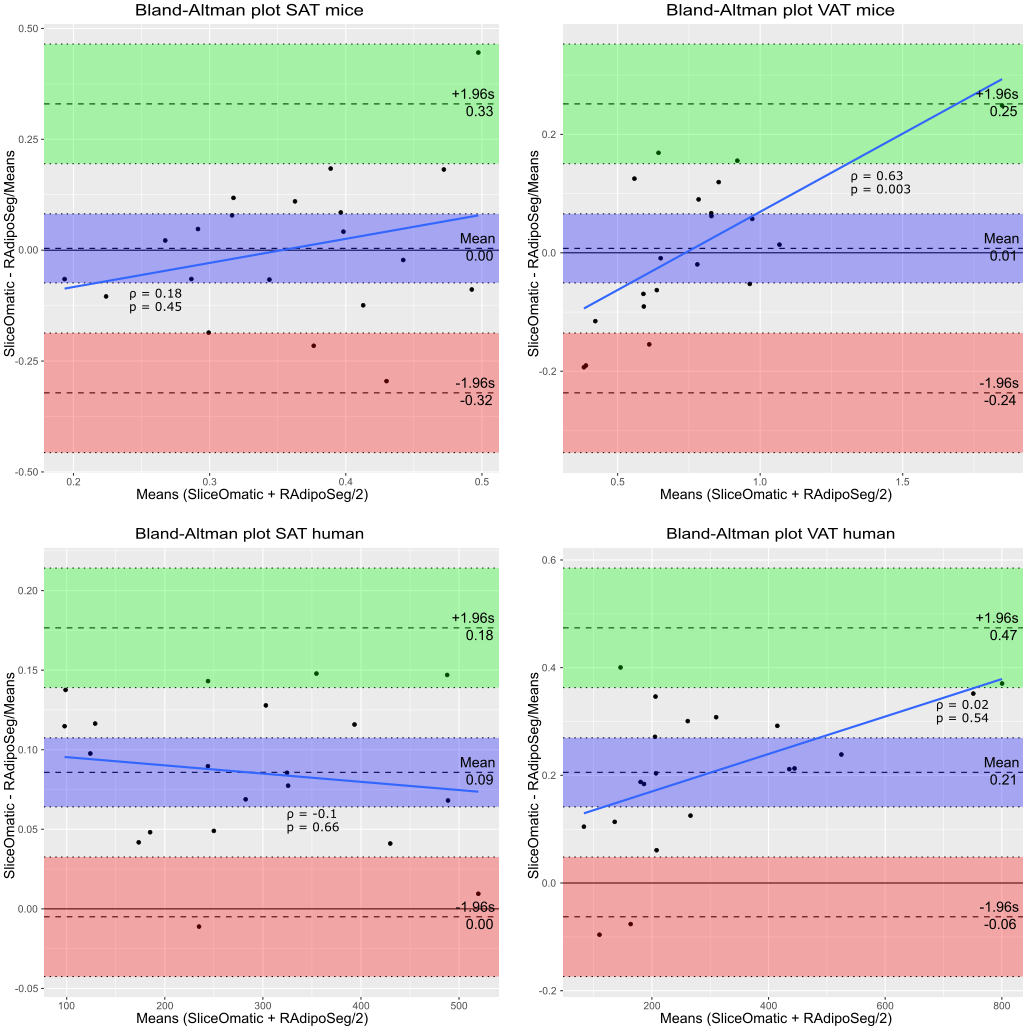

Supplement: Supplementary file 4 — Additional file 4. Bland-Altman plots with proportions on the y-axes. Plots calculated with volume in cm3 of VAT and SAT for mice (n=20) and humans (n=20), with 1.96 ×SD limits of agreement and 95 % confidence interval. Volumes were calculated by adding the voxels from all images of each subject and multiplying with the voxel size. Data from the lean and obese mice were pooled together. Spearman’s rank correlation coefficients were calculated for estimation of proportional bias. [file 13098_2022_913_MOESM4_ESM.png]

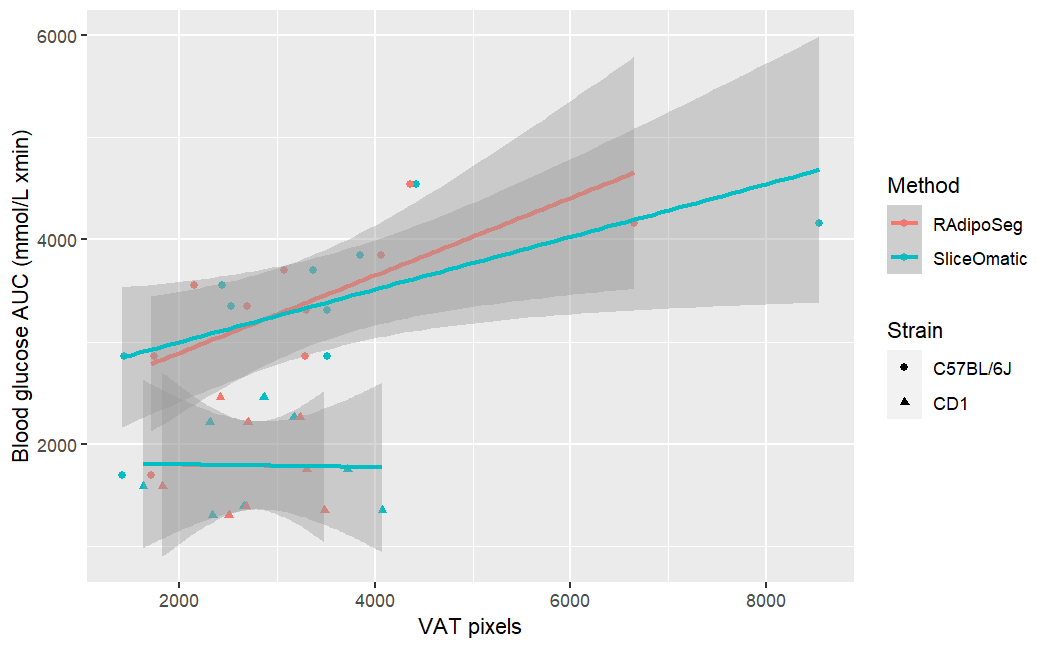

Supplement: Supplementary file 5 — Additional file 5. Blood glucose AUC plotted against VAT volume. The plot was separated by methods, RAdipoSeg and Sliceomatic, and mouse strain, CD1 and C57BL/6J. Test of linear correlations were performed using Spearman’s rank correlation coefficients. P-values (rho) for the CD1 mice were 0.84 (− 0.10) for RAdipoSeg and 0.93 (0.05) for SliceOmatic. P-values (rho) for the C57BL/6J mice were 0.01 (0.79) for RAdipoSeg and 0.01 (0.78) for SliceOmatic. [file 13098_2022_913_MOESM5_ESM.png]
